# Probing cytochrome *c* in living mitochondria with surface-enhanced Raman spectroscopy

**DOI:** 10.1038/srep13793

**Published:** 2015-09-08

**Authors:** Nadezda A. Brazhe, Andrey B. Evlyukhin, Eugene A. Goodilin, Anna A. Semenova, Sergey M. Novikov, Sergey I. Bozhevolnyi, Boris N. Chichkov, Asya S. Sarycheva, Adil A. Baizhumanov, Evelina I. Nikelshparg, Leonid I. Deev, Eugene G. Maksimov, Georgy V. Maksimov, Olga Sosnovtseva

**Affiliations:** 1 Department of Biophysics, Biological Faculty, Moscow State University, Leninskie gory 1/12, Moscow, 119234, Russia; 2 Department of Biomedical Sciences, Faculty of Medical and Health Sciences, Copenhagen University, Blegdamsvej 3, Copenhagen, DK-2200, Denmark; 3 Laser Zentrum Hannover e. V., Hollerihallee 8, D-30419 Hannover, Germany; 4 Department of Technology and Innovation, University of Southern Denmark, Odense M, DK-5230 Denmark; 5 Department of Nanomaterials, Faculty of Material Sciences, Moscow State University, Moscow, Leninskie gory 1/73, 119991, Russia; 6 Department of Inorganic chemistry, Faculty of Chemistry, Moscow State University, Moscow, Leninskie gory 1/3, 119991, Russia; 7 Kurnakov Institute of General and Inorganic Chemistry of Russian Academy of Sciences, Moscow, Leninskiy prospekt, 119992, Russia

## Abstract

Selective study of the electron transport chain components in living mitochondria is essential for fundamental biophysical research and for the development of new medical diagnostic methods. However, many important details of inter- and intramembrane mitochondrial processes have remained in shadow due to the lack of non-invasive techniques. Here we suggest a novel label-free approach based on the surface-enhanced Raman spectroscopy (SERS) to monitor the redox state and conformation of cytochrome *c* in the electron transport chain in living mitochondria. We demonstrate that SERS spectra of living mitochondria placed on hierarchically structured silver-ring substrates provide exclusive information about cytochrome *c* behavior under modulation of inner mitochondrial membrane potential, proton gradient and the activity of ATP-synthetase. Mathematical simulation explains the observed enhancement of Raman scattering due to high concentration of electric near-field and large contact area between mitochondria and nanostructured surfaces.

Mitochondria are organelles of fundamental importance for cellular energy production, metabolic regulation, aging and cell survival under stress^1^,^2^,^3^. Normal function of mitochondria and their pathological changes, including production of reactive oxygen species (ROS), are heavily dependent on the redox state of the electron transport chain (ETC) cytochromes and cytochrome *c* in particular^4^,^5^. At present, most of the studies of isolated mitochondria and mitochondria in cells are performed by fluorescent microscopy, absorption spectroscopy and measurements of O_2_ consumption^3^,^6^,^7^,^8^. The fluorescent microscopy with small fluorescent dyes (rhodamin and MitoTracker-family, etc.) or fluorescent proteins (GFP, YFP, RFP) can provide general information about changes in the potential of the inner mitochondrial membrane (ΔΦ), the mitochondrial volume, and the co-localization of certain mitochondrial components with a molecule of interest^3^. In spite of numerous advantages, these methods provide only indirect information about the redox state of ETC complexes. Registration of autofluorescence of NADH and FAD^+^ allows to estimate the redox state of complexes I and II^9^ but this approach does not work for complexes III and IV or cytochrome *c.* Measurements of the fluorescence of ROS-selective dyes provide overall information about ETC overflow with electrons^10^. Indirect estimation of the redox state of cytochromes *c* and *b*-type was achieved by either absorption spectroscopy or measurements of O_2_ consumption by the complex IV in isolated inner mitochondrial membrane (IMM) species with an externally added cytochrome *c*. The direct information about cytochrome *c* redox state and its intermembrane space (IMS) dynamics in living mitochondria is still difficult to obtain because the redox state and the conformation of cytochrome *c* are highly dynamic, affecting its diffusion in IMS, interaction with complexes III and IV and the electron transfer^1^,^11^,^12^. Thus, sensitive, noninvasive, label-free analysis of cytochrome *c* inside intact functional mitochondria can extend our understanding of cytochrome *c* role in the modulation of ETC activity and development of mitochondria pathologies.

Raman scattering of cytochromes is sensitive to their redox state, mitochondrial membrane potential and to the activity of electron transport^13^,^14^. This makes Raman spectroscopy a promising tool for non-invasive studies of the electron transfer in mitochondria. However, Raman scattering of oxidized cytochromes has much lower intensity than scattering of reduced forms^13^,^16^,^17^,^18^. Therefore label-free monitoring of cytochromes in intact mitochondria or living cells and organs can be achieved preferentially for reduced forms in the case of conventional resonance or non-resonance Raman spectroscopy^15^,^16^,^17^,^18^. All previous Raman studies of isolated mitochondria and isolated cytochrome-containing ETC complexes were performed using artificially reduced samples^13^,^14^. Since in functional mitochondria all ETC complexes, including cytochromes, are in a partially reduced state^1^,^16^,^17^,^18^, the enhancement of Raman scattering from oxidized cytochromes in mitochondria seems to be an important task.

In surface enhanced Raman spectroscopy (SERS), Raman scattering can be enhanced by many orders of magnitude if probed molecules are located near silver or gold nanostructures^19^,^20^,^21^,^22^,^23^. Previously, SERS spectra were recorded from purified oxidized or reduced cytochrome *c* adsorbed on silver electrode or mixed with silver colloid^19^,^24^. Both reduced and oxidized cytochromes possess intensive SERS spectra and can be distinguished from one another. Previously this cytochrome feature was used for the development of O_2_-sensitive SERS-probe based on gold nanoparticles with the attached cytochrome *c*^25^. However, to the best of our knowledge, SERS has never been used to monitor redox states and conformation of cytochromes in their natural environment—in ETC of living mitochondria.

We present novel results of SERS study of cytochrome *c* in living functional mitochondria placed on hierarchically structured silver surfaces (AgNSSs). We demonstrate that SERS spectra of cytochrome *c* in mitochondria exhibit changes under dissipation of the proton gradient across the IMM and under the inhibition of ATP synthesis. We employ mathematical simulation of focusing of evanescent waves by silver nanoparticles to explain the long-distance enhancement of Raman scattering from cytochrome *c* in mitochondria.

## Results

### SERS features of living mitochondria

**Figure 1:**
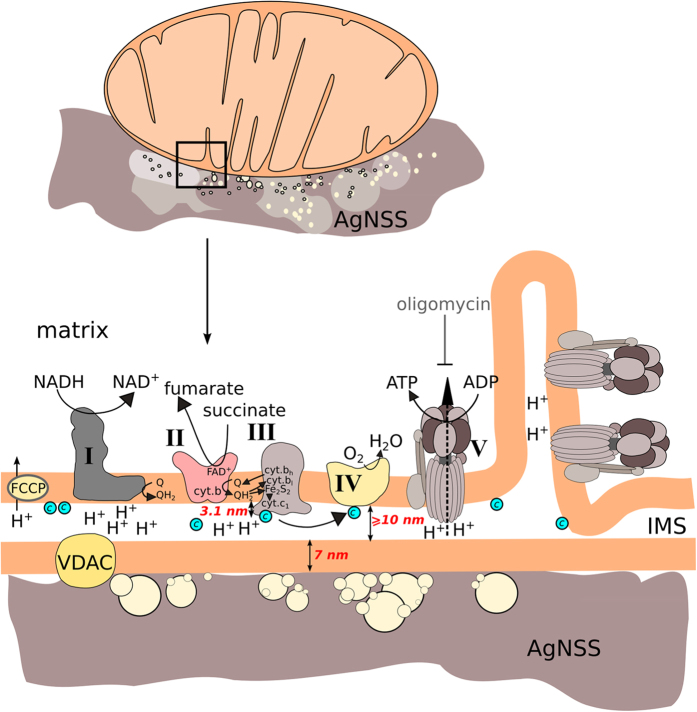
Scheme of mitochondria placed on silver nanostructured surface (AgNSS). Schematic presentation of the magnified view of the outer mitochondrial membrane in a contact with AgNSS, intermembrane space (IMS) and internal mitochondrial membrane with complexes of the electron transport chain. Cytochromes of *b* and *c*-type are shown in corresponding complexes. Cytochrome c is shown as cyan ball. Red numbers indicate the approximate height of the outer mitochondrial membrane, size of IMS and IMS domain of complex III (data on distances and sizes of mitochondrial elements are taken from^11^,^29^). VDAC–voltage-dependent anion channel; FCCP—protonophore (Carbonyl cyanide-*p*-trifluoromethoxyphenylhydrazone) used in the study.

ETC of mitochondria includes cytochromes of *b*- (in complex II and III), *c*- (in complex III and cytochrome *c*) and *a*-types (in complex IV) (Fig. 1). Under used experimental conditions (532 nm laser), Raman scattering could be excited only from cytochromes of *c*- and *b*- types.

**Figure 2:**
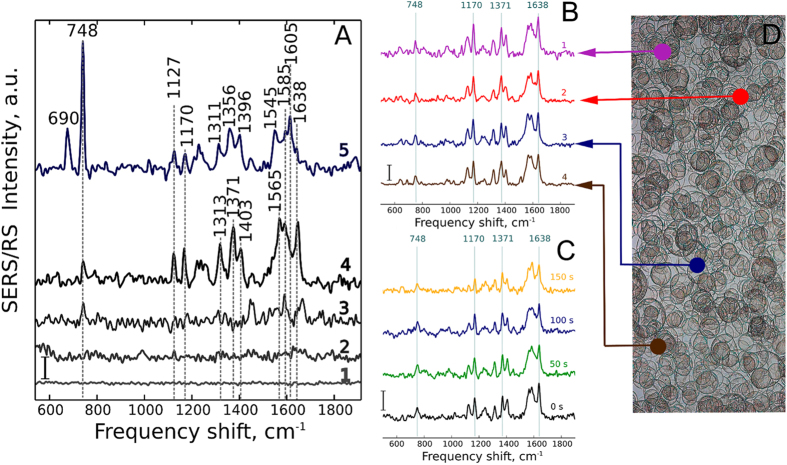
SERS spectra of mitochondria. (**A**) (1) Spectrum of mitochondrial buffer on AgNSS; (2) spectrum of mitochondria suspension used in SERS experiments placed into the Petri dish with smooth silver plate without nanostructures; (3) Raman spectrum of concentrated mitochondrial aggregate placed into ordinary Petri dish; excitation power 1.5 mW, objective x63, NA 0.9, registration time 20 s; (4) SERS spectrum of mitochondria suspension placed on AgNSS after pyruvate, succinate, ADP and MgCl_2_ application; (5) SERS spectrum of mitochondria suspension placed on AgNSS after application of sodium dithionite. SERS spectra were recorded from the diluted mitochondria sample with excitation power 1.5 mW; objective x5, NA 0.15, registration time 20 s. (**B**) SERS spectra of mitochondria recorded from different places of AgNSS shown schematically by colored spots in Fig. D. (**C**) SERS spectra of mitochondria recorded from the same place on AgNSS with time lapse between spectra acquisition of 30 s. Accumulation time for each spectrum is 20 s. Dotted vertical lines indicate positions of maxima of the most intensive peaks. Vertical scale bars are equal to 200 a.u in all figures. (**D**) Optical microphotograph of Ag nanostructured surface in the transmitted light. Horizontal length of the microphotograph is 100 μm. Detailed morphology of AgNSSs is shown in Fig. 4.

In our experiments SERS spectra of mitochondria placed on AgNSS demonstrate a set of intensive peaks corresponding to heme molecules of cytochromes of mitochondrial ETC with the position of their main maxima at 748, 1127, 1170, 1313, 1371, 1565, 1585 and 1638 cm^−1^ (Fig. 2A (spectrum 4), Table 1). These peaks originate from the normal group vibrations of pyrrol rings, methine bridges and side radicals in the heme molecule (Fig. 2, Table 1, Supplementary Fig. 1). Similar spectra of the same shape and with the similar set of peaks are observed from purified oxidized cytochrome *c* mixed with Ag colloid (Supplementary Fig. 2A). Note, that SERS spectra of mitochondria show a peak at 1313 cm^−1^ as a signature of the *с*-type cytochrome and do not have peaks at 1300 and 1337 cm^−1^ which are specific of *b*-type cytochromes, myoglobin and hemoglobin (Fig. 2A, Supplementary Fig. 2)^18^,^19^,^24^. This is a clear evidence of the origin of SERS spectra of mitochondria from the *c*-type heme in cytochrome, namely, the cytochrome *c*. It is likely that the heme of cytochrome *c1* (which is located further from outer mitochondrial membrane than cytochrome *c* and from AgNSS surface) does not contribute much to the SERS spectra of mitochondria. Cytochromes *b* of the complexes II and III are localized in the IMM part, resulting in no enhancement of their Raman scattering due to a too large distance between *b*-hemes and the AgNSS surface. To record SERS spectra of mitochondria with fully reduced electron carriers we added sodium dithionite (SDT) to the mitochondria sample. SERS spectra of SDT-treated mitochondria correspond to that of reduced purified cytochrome *c* and demonstrate a signature shift of positions of peaks sensitive to the redox state of Fe atom (Fig. 2A (spectrum 5), Table 1, Supplementary Fig. 2A). Additionally, we observed increased relative intensity of the peak at 748 cm^−1^ in the SERS spectra of SDT-treated mitochondria and reduced cytochrome *c* (Fig. 2A (spectrum 5), Supplementary Fig. 2A). Such an increase is already known for Raman spectra of reduced cytochromes, SDT-treated mitochondria, cardiomyocytes and heart^16^,^17^,^18^ and for SERS spectra of reduced cytochrome *c*^19^. The same mitochondrial sample placed into the Petri dish with a smooth silver plate on the bottom did not give any spectrum (Fig. 2A, spectrum 2). We could obtain ordinary Raman spectra of intact mitochondria only by focusing laser light inside dense mitochondria aggregates using much higher laser power per area (objective x63, NA 0.9 and laser power 1.5 mW per registration spot with a diameter of 400 nm) (Fig. 2A, spectrum 3). Notably, even under such high excitation power ordinary Raman spectrum of mitochondria is noisy and does not contain Raman peaks of cytochromes. Only two weak peaks of protein bond vibrations can be observed around 1450 and 1660 cm^−1^ (corresponding to C-H deformations and amide bond vibration, respectively). Interestingly, we never observed SERS spectra of purified cytochrome *c* (in the concentration range of 10^−5^–10^−7^ M) placed on AgNSS, while mitochondria gave intensive SERS spectrum almost immediately after placement of the sample into the dish with AgNSS. SERS spectra of mitochondria recorded from different AgNSS spots and from the same AgNSS spot over time are highly reproducible (Fig. 2B,C). Stability of mitochondria SERS spectra recorded from the same spot at different time points indicates absence of mitochondria photodamage under laser illumination. To check excitation condition that can cause sample damage we intentionally increased laser power. We observed photodamage-induced broadening of mitochondria SERS peaks only under 10-fold higher excitation power, than that used in all described SERS experiments (Supplementary Fig. 3).

Importantly, AgNSSs themselves did not affect integrity and function of mitochondria. Intactness of mitochondria was determined using protonophore FCCP to check coupling between the electron transport and ATP synthesis. The coupling exists only in mitochondria with intact membranes and therefore mitochondria response to FCCP is a reliable test on their integrity. It is known from the literature, that FCCP uncouples the electron transport and ATP synthesis in mitochondria, causing dissipation of H^+^-gradient across IMM and IMM depolarization, thus increasing the electron transport rate in ETC to recover H^+^-gradient and ΔΦ^26^,^27^. This leads to decrease in the relative amount of reduced electron carriers and reduced electron donors^17^. To estimate the effect of FCCP on mitochondria we used two techniques: registration of excitation spectra of mitochondria autofluorescence at 490 nm (corresponding to NADH fluorescence) and registration of O_2_ consumption by mitochondria. We compared responses to FCCP for control mitochondria and mitochondria placed on AgNSS and illuminated by green laser. Application of FCCP (1 and 20 μM) to mitochondria decreased the amount of NADH (its reduced form which is used as the initial electron donor in the complex I) in both control mitochondria and mitochondria placed on AgNSS and illuminated by green laser light (for details see Supplementary Information and Supplementary Fig. 4). Application of ADP after succinate and pyruvate to all mitochondria samples similarly increased oxygen consumption, which was additionally strongly increased by the following FCCP (20 μM) application (Supplementary Table 1). The obtained results demonstrate a pronounced response of mitochondria to the FCCP-induced disruption of H^+^-gradient. This indicates existence of mitochondrial H^+^-gradient (both in experimental buffer and under conditions of SERS experiment) before FCCP application and that electron transport and ATP synthesis are coupled. Such a response is possible only in mitochondria with intact membranes.

**Figure 3:**
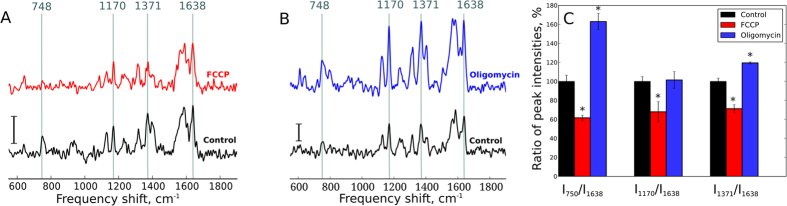
Dependence of SERS spectra of mitochondria on proton-motive force and activity of ATP synthesis. (**A**) SERS spectra of mitochondria before and after application of FCCP (0,5 μM) (lower and upper spectra, respectively). (**B**) SERS spectra of mitochondria before and after application of oligomycin (10 μM) (lower and upper spectra, respectively). (**C**) Ratios of peak intensities calculated for SERS spectra of mitochondria in control (after application of pyruvate, succinate, ADP and MgCl_2_, black bars) and in 2 min after application of protonofor FCCP (0,5 μM) (red bars, number of experiments n = 3) or oligomycin (10 μM) (blue bars, number of experiments n = 3). Ratios were calculated by dividing intensities of peak maxima at 750 by 1638 cm^−1^ (I_750_/I_1638_), 1170 by 1638 cm^−1^ (I_1170_/I_1638_) and 1371 by 1638 cm^−1^ (I_1371_/I_1638_). Ratios in control measurements were taken as 100%. Vertical bars show SE value, %. SE value of control ratios were calculated from 10 independent measurements of control SERS spectra from the same spot.

To demonstrate high sensitivity of SERS spectra of mitochondria to the proton gradient across IMM and mitochondrial membrane potential (ΔΦ) and to the redox state of other ETC complexes, we applied (i) protonophore FCCP and (ii) oligomycin as an inhibitor of ATP-synthetase (ETC complex V). As it was mentioned, FCCP-induced uncoupling of the electron transport and ATP synthesis leads to the decrease in the relative amount of reduced electron carriers including cytochrome *c*^17^. Oligomycin, on the contrary, due to the inhibition of ATP-synthase, causes accumulation of protons in the intermembrane space causing IMM hyperpolarization, decrease in the electron flow rate and the increase in the relative amount of the reduced electron carriers in ETC. As expected, we observed a decrease in the relative normalized intensity of cytochromal peaks at 748, 1127, 1170 and 1371 cm^−1^ under the FCCP (0.5 μM) treatment (Fig. 3A,C). The application of oligomycin (10 μM), oppositely, caused an increase in the relative input of peaks at 748 and 1371 cm^−1^ into the overall spectrum that can be seen as the increase in relative intensities of peaks at 748 and 1371 cm^−1^ normalized to the intensity of the peak at 1638 cm^−1^ (ratios I_748_/I_1638_ and I_1371_/I_1638_) (Fig. 3B,C). Importantly, that the observed effect of FCCP and oligomycin on SERS spectra was not associated with the direct interaction of FCCP or oligomycin with cytochrome c molecules in mitochondria, since both chemicals did not affect SERS spectra of purified cytochrome *c* (Supplementary Fig. 5). We conclude that the behavior of *c*-type cytochrome SERS peaks following FCCP and oligomycin treatments is in excellent agreement with the expected dependence of the reduction state of *c*-type cytochromes on the proton-motive force and the ETC loading with electrons.

### Long-distance Raman enhancement

It is commonly believed that surface enhancement of Raman scattering of molecules occurs in an extremely close proximity to NPs or nanostructured surfaces^20^,^28^. However, our experiments demonstrate that AgNSSs cause the enhancement of Raman scattering of *c*-type cytochromes in intact mitochondria. Cytochrome *c* is known to exist in several fractions: (i) immobilized pool, bound to cardiolipin on the surface of internal mitochondrial membrane, (ii) mobile pool—3D and 2D-diffusing cytochrome molecules moving in the intermembrane space near or between the inner and outer mitochondrial membranes and randomly interacting with ETC complexes III and IV and (iii) cytochrome molecules short-term bound to the complexes III or IV accepting or donating electrons (Fig. 1)^11^,^12^. Mitochondria are very dynamic: the distance between outer and inner membranes varies with many factors, including matrix volume, activity of ATP synthesis and IMS amount of ADP, the number of contact sites between outer and inner mitochondrial membranes, etc.^11^,^12^,^29^,^30^,^31^. Therefore, it is hard to estimate precise distances between membranes, outer membrane and diffusing cytochrome *c* and cytochrome *c* bound to complex III and mitochondria surface. According to the literature^11^,^29^,^30^,^31^,^32^, the mitochondrial membrane thickness is about 7 nm, intermembrane space thickness exceeds 10 nm and the intermembrane part of complex III is around 3 nm (we should note that these distances can change under variation of ETC activity). Thus, under the ETC function the distance between nanostructures of AgNSS and cytochrome *c* in mitochondria varies between 7–17 nm and can be bigger if the intermembrane space thickness increases. The distance between cytochrome c bound to complex III and AgNSS is at least 13 nm (Fig. 1). Cytochrome *c*, bound to IMM or complex IV locates at appr. 17 nm from AgNSS (Fig. 1). Relying on these data, we suppose that the SERS signal comes from 3D-diffusing cytochrome *c* and cytochrome *c* bound to complex III, while cytochrome *c* interacting with complex IV or cardiolipin of IMM does not contribute to SERS spectra. This assumption is based on the fact that cytochromes *b* in the complex III are located closely to the intermembrane surface of IMM and we do not see any cytochrome *b*-specific peak in SERS spectra of mitochondria. For better understanding of this long-distance enhancement of Raman scattering from cytochrome *c* in mitochondria, numerical simulation of the electric near field on the nanostructured silver surface under different illumination conditions was performed (see below subsection “Mathematical simulation”).

### Characterization of AgNSS structure

**Figure 4:**
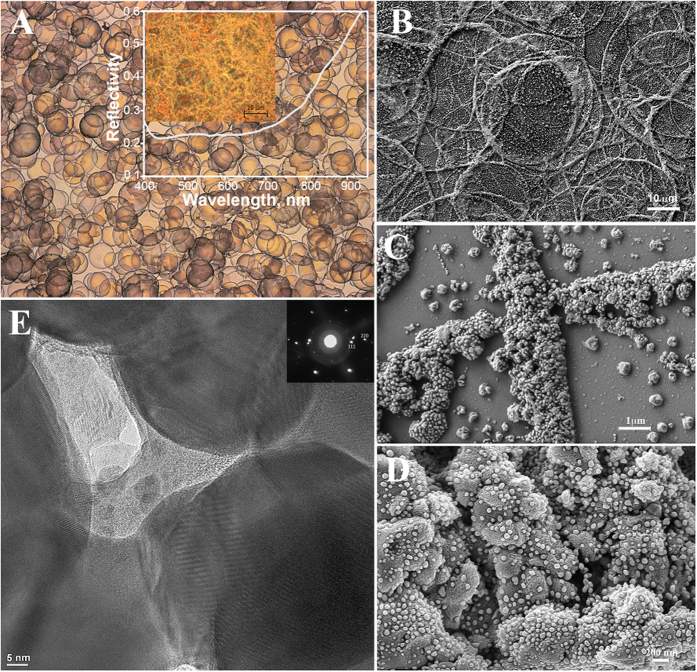
Characterization of Ag nanostructured surfaces. (**A**) Optical microphotograph of Ag nanostructured surface in the transmitted light. The inset figure shows AgNSS in reflected light and the reflectivity spectrum of AgNSS obtained with x50 magnification, objective NA 0.75. (**B**–**D**) Scanning electron microscopy images of Ag nanostructured surface with different magnifications; white horizontal scale bars are equaled to 10, 1 and 0.2 μm, respectively. Figure B shows overlapping of nanostructured silver rings. Figure C demonstrates hierarchically organized clusters (“bricks”) forming concaved walls of nanostructured silver rings. Figure D shows magnified view of porous nanostructured silver “bricks” covered with smaller spherical silver nanoparticles. Figure E demonstrates TEM image of the hierarchically structured silver showing channels in the porous silver bricks. The channels are filled with nanometer—sized embryocrystals of silver formed by fast thermal decomposition of diaminsilver hydroxide solution from initially sprayed aerosol droplets. The embryocrystals are being moved through the channels onto the surface of porous silver bricks due to capillary forces followed by evaporation of water solvent and the crystal overgrowth up to the sizes of “sesame seeds”. White horizontal scale bar is equaled to 5 nm.

To build a model of Raman enhancement of mitochondrial cytochrome *c* on AgNSSs, we performed their characterization with scanning and transmission electron microscopy combined with local reflectance spectra recording. The obtained AgNSS have highly overlapping silver rings of a complex morphology with multilevel arrangement of AgNPs (Fig. 4A). The AgNSS structure originates from the decomposition of micrometer-sized droplets of ultrasonic mist of diaminsilver (I) hydroxide solution, this phenomenon is known as the coffee-ring effect^33^. The silver ring formation is accompanied by multiscale structuring due to complex redistribution of nutrient liquid and building block overgrowth (Fig. 4B–D). During silver reduction, larger and smaller gaps appear in the region of ring rims resulting in randomly organized blocks (‘‘bricks of the wall’’) of 200–500 nm (Fig. 4B–D). “Blocks” of highly overlapping rings are covered with interconnected silver particles of 10–50 nm where smaller AgNPs lying over larger AgNPs like sesame seeds resulting in the multiscale structure of AgNSS (Fig. 4D). We consider that the porosity feature and capillary effects initiates the growth of the “sesame seeds” which eventually coveri the surface of silver rings in many points (Fig. 4E). The reflectivity spectra recorded from various parts of AgNSS using x50 magnification are similar to one another and have a wide reflectance dip in the region of 400–850 nm (Fig. 4). The light absorption at a wide wavelength range can be explained by a complex multiscale structure of AgNSS. The similarity of reflectance spectra recorded from spots with various magnifications (data are not shown) is an evidence of AgNSS microscopic homogeneity that is highly important for serial biomedical studies.

Importantly, the above mentioned AgNSS structure was not affected by mitochondria in the physiological buffer and by laser illumination during experiments. To test the effect of preparation on AgNSS after the SERS experiment we placed AgNSSs into the glass with MilliQ water to remove mitochondria and buffer salts. After two hours of incubation in MilliQ water we dried AgNSSs and performed EDX and SEM measurements. EDX/SEM results demonstrate no essential interaction of AgNSS with components of the physiological buffer and mitochondria (Supplementary Fig. 6). In particular, chlorine and sulfur relative concentrations do not exceed 0.3–0.5 %. There is also no signature of silver recrystallization or degradation as the components of AgNSS remain of 30–150 nm size with the distinctive multiscale structure. The absence of interaction of these particular AgNSS with physiological buffer or mitochondria under laser irradiation is a key moment providing reproducible SERS spectra.

We assume that the enhancement of Raman scattering of mitochondrial cytochromes is due to the unique “sesame seed” multiscale structure of AgNSSs. Such a hierarchic structure of AgNPs (Fig. 4) can result in (i) a significant amplification of electric fields near AgNSS, (ii) in better immobilization of mitochondria on AgNSS, and (iii) in the higher number of contact sites between mitochondria with AgNSS. To verify this hypothesis we synthesized simpler nanostructured surfaces consisting of several layers of spherical nanoparticles with a diameter of 10–50 nm without a hierarchic structure (Suplementary Fig. 7). We could not record any SERS spectra from mitochondria placed on such surfaces.

### Mathematical simulation

Our explanation of the observed surface-enhanced Raman scattering from intact mitochondria is based on several known facts related to the problem of light scattering by metal nanoparticles located near a metal surface: (i) if the size of nanoparticles is much smaller than the wavelength of incident light, the nanoparticle can be considered an electric dipolar scatterer with a corresponding dipolar polarizability^34^, and (ii) the induced dipole moment of this nanoparticle is determined by the incident and reflected electric fields and by nanoparticle-surface interaction which can be estimated using the method of images^35^. Following this method, dipole moment vectors of the real electric dipole and its image have opposite directions, when the real electric dipole aligned in parallel to the surface and the same directions in the case of perpendicular alignment^36^. Due to such dipole-surface interaction, the in-plane components of the dipole moment are decreased and the normal component is increased. Thus, if the induced electric dipole moment of the nanoparticle located on metal surface has mainly the normal component, a strong electric near field enhancement can be produced.

**Figure 5:**
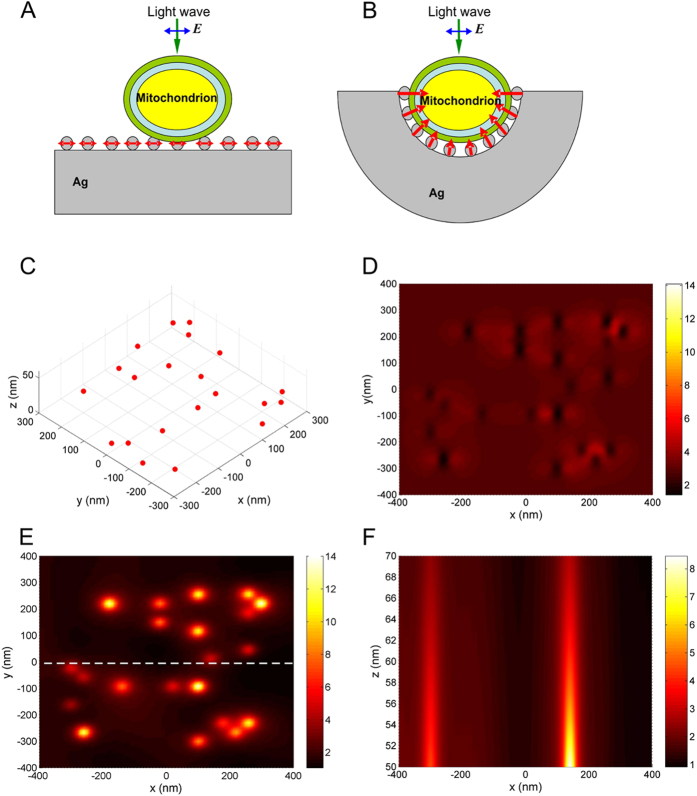
Results of mathematical simulation. Schematic presentation of a mitochondrion located: (**A**) on a flat Ag nanostructured surface with Ag nanoparticles and (**B**) in a cavity on Ag nanostructured surface. Mitochondrion is illuminated by linear-polarized light wave. Blue arrows show polarization of the electric field *E*. Red arrows indicate: (**A**) induced total dipole moment of Ag nanoparticles, and (**B**) normal component (with respect to the substrate surface) of the light-induced dipole moment of Ag nanoparticles. (**C**) Distribution of Ag nanoparticles with diameters of 40–50 nm on Ag surface used in numerical simulations. (**D**) Electric field intensity calculated in a plane, 60 nm above the Ag surface (XY-plane), when the structure is normally irradiated by linear-polarized plane light wave with the wavelength of 532 nm. (**E**) Electric field intensity calculated in a plane, 60 nm above the Ag surface, when the structure is irradiated at 65 degrees by TM-polarized plane light wave with the wavelength of 532 nm. (**F**) Distribution of the electric field intensity (corresponding to the case (**E**)) in the plane perpendicular to the Ag surface. The plane passes through the dashed line shown in (**E**).

The multiscale inhomogeneous distribution of nanoparticles in the AgNSSs enables excitation of strong electric near-fields in the system. In the experiments external light is normal incident with respect to the sample. However, because many nanoparticles of the AgNSS are located on side walls of different cavities (Fig. 4C,D), they are irradiated by polarized light under the incline conditions with respect to the surface. As a result, the light electric field directed perpendicular to the side walls of the cavities will induce strong nanoparticle’s electric dipole moments. This will lead to induction of strong nanoparticle's electric dipole moments by the light electric field directed perpendicular to the side walls of the cavities. Our approach to explain the observed strong Raman scattering is illustrated schematically in Fig. 5A,B. There are two different possibilities. When a mitochondrion is located on a flat metal surface covered by nanoparticles and is irradiated by normally incident light (Fig. 5A), the mitochondrion has a limited contact area interacting with weak optical near fields generated by the in-plane induced dipole moments (red arrows in Fig. 5A). In this case the Raman signal has to be low. The Raman signal should be much stronger if the mitochondrion is located within a cavity with walls covered by nanoparticles, as in the multiscale AgNSSs (Figs 4 and 5B). Much stronger optical near fields must exist due to the light-induced normal components of the nanoparticle electric dipoles. The distribution of these electric fields can be imagined as electric-field “needles” deeply penetrating into the mitochondrion (Fig. 5B). In this case, the contact area between the mitochondrion and nanoparticle structure is also increased providing better conditions for Raman scattering. We assume that this situation corresponds perfectly to our experimental conditions.

In order to support these qualitative conclusions, we tested a simple physical model including randomly distributed silver nanoparticles located on a flat silver surface. Numerical simulations of the electric field intensity in the near-field region above the silver nanoparticles are performed under different irradiation conditions. Incline illumination of nanoparticles in the model corresponds to the case when nanoparticles in the AgNSS are located on side walls of the larger-scale cavities and illuminated by external light. We demonstrate numerically that the strong electric near-field can be realized in a system of silver nanoparticles located on silver substrate under incline illumination conditions by light with transverse magnetic field polarization (TM-polarization). In real systems, it corresponds to realization of strong electric near-fields inside the cavities with nanoparticles (Fig. 4D). Results of numerical simulations are presented in Fig. 5C–F. Positions of Ag nanoparticles with diameters of 40–50 nm placed on the flat Ag surface (XY- plane) are shown in Fig. 5C. When the nanoparticle structure is irradiated by normally incident linear-polarized light beam, the electric near field above nanoparticles is very weak (Fig. 5D). When the same structure is irradiated at 65 degrees by TM-polarized light, the electric near-field above the nanoparticles increases significantly due to the presence of normal induced dipole components (Fig. 5E,F). In the plane perpendicular to the substrate surface, the near-field generated by every dipole looks like the mentioned above electric field needles (Fig. 5F). This corresponds to our expectations: a strong contribution to the enhancement of Raman scattering is due to nanoparticles that are located on side walls of cavities of multiscale AgNSSs (Fig. 4D) which are illuminated by light of certain polarization with respect to the cavity surfaces. Note that in our numerical simulations the incident angle of 65 degrees is chosen merely as an example. In the real situation various incident conditions for light interacting with nanoparticles are realized due to the random configurations of cavities in AgNSSs. The incident angle and light polarization can change within a wide range with the respect to the side walls of irregular cavities in AgNSSs. As a result, in AgNSS there always exist many regions where the considered mechanism of electric near-field enhancement is realized providing strong Raman signals for mitochondria.

## Discussion

In this paper we present the first SERS study of functional intact mitochondria performed using silver nanostructed surfaces. Mathematical simulation confirmed that light-induced excitation of strong electric near-fields arise from the complex multi-level morphology of AgNSSs. These fields and a large contact area between mitochondria and nanoparticles are responsible for strong enhancement of Raman scattering of cytochrome *c* in ETC of intact mitochondria. Our numerical model suggests that direct interactions between nanoparticles are not important^37^. Note that so far we did not take into account surface plasmon resonances (SPR)^38^,^39^. Spectral positions of SPR are dependent on nanoparticle size, shape and environment^38^. Due to complex multiscale properties of the AgNSSs the SPR of certain nanoparticles can be excited by light used in our experiments resulting in stronger enhancement of electric near-fields in the system. Another possible mechanism of electric near-field enhancement in the system is surface plasmon polaritons^40^, that can additionally contribute to the increase in the intensity of Raman scattering together with the described above. Propagating surface electromagnetic waves (surface plasmon polaritons) can be excited at the AgNSSs by light incident at practically any angle via scattering by nanostructures^41^. Owing to the exponential localization of surface plasmon polaritons (SPPs) near a metal surface, their electric fields are localized in the near-field zone above the surface, extending over 100 nm away from the surface plane. The corresponding electromagnetic enhancement factor of Raman scattering thereby decays exponentially over 25 nm distance from the surface. Multiple scattering of SPPs by surface roughness and different nanoparticles of a complex metal structure can result in the spatial (strong) localization of SPPs in the surface plane^42^, and in creation of the SPP “hot spots”, where the density of electromagnetic energy is significantly increased. If mitochondria end up in the regions with such “hot spots” the Raman signal of cytochrome *c* diffusing in IMS and, possible, cytochrome *c* bound to complexes III and IV will be significantly increased.

At present there are only few SERS studies of cells or cell organelles done by means of nanostructured surfaces^33^,^43^,^44^. Most of fundamental and diagnostically-oriented studies of cells and isolated biomolecules are done by SERS with silver or gold colloids^21^,^22^,^23^,^24^,^25^,^45^,^46^,^47^,^48^. However, they can be cell toxic, can change osmolarity of the cell environment, distribute inside the cell or on the cell surface non-evenly and can aggregate altering Raman enhancement^47^,^49^,^50^. In this sense, nanostructured surfaces have several advantages, since they are more stable, do not contain synthesis by-products and can give reproducible Raman enhancement. Another advantage of our study is that we performed SERS measurements of cytochrome *c* in living mitochondria without direct contact of cytochrome *c* with Ag nanostructures. This situation is much more preferable, than direct interaction of an analyte molecule with the plasmonic nanoparticle, because of a possibility of nanoparticle-induced changes in the molecule conformation followed by possible alteration of the molecule function^45^.

Isolated mitochondria are widely used objects in biomedical studies^1^,^2^,^4^,^5^,^6^,^7^,^11^,^12^. Despite the number and diversity of the mitochondria research techniques, there still is a lack of methodological approach to perform selective study of cytochrome c redox and conformational changes in ETC of living mitochondria. There were two reports on SERS and TERS observation on isolated mitochondria^51^,^52^. In the first study Karatas and co-authors observed SERS spectra of lipids, proteins and glycosaccharides in mitochondria mixed with gold colloidal suspension^51^. They did not observe any peaks from heme of cytochromes, could not characterize cytochromes and, therefore, electron transport in ETC of mitochondria. Bohme and co-authors performed TERS study on dried mitochondria and observed two non-intensive peaks around 1360 and 1620 cm^−1^ that they attributed to the heme of cytochrome *c*^52^. However, drying of the mitochondria prevented the possibility to study cytochrome *c* function in ETC under electron transfer. For the first time we demonstrate that by means of specially designed AgNSS it is possible to enhance Raman scattering of *c*-type cytochromes in ETC of functional living mitochondria and that SERS spectra of mitochondrial cytochrome *c* are sensitive to the H^+^-gradient across IMM, mitochondrial ΔΦ and ATP synthesis by ATP synthase. The limitation of the proposed approach is that it can be only used to study cytochrome *c* in isolated mitochondria, not in mitochondria in cells, since nanostructured surfaces are not endocytosized. However, we believe that presented results can be used for further development of internalized nanoparticles with a special shape ensuring enhancement of Raman scattering from cytochromes in mitochondria *in situ*.

To conclude, the enhancement of Raman scattering from cytochrome *c* in intact mitochondria is of special interest since its redox state and conformation can both affect and be influenced by the overall state of mitochondria ETC, lipid composition of mitochondrial membrane, apoptosis-related processes, and damage of ETC by ROS. Our data suggest that the proposed SERS-based approach can be used to study conformational and redox state changes in cytochrome *c* in respiring isolated mitochondria under various conditions.

## Materials and Methods

### Preparation of silver nanostructured surfaces

Silver nanostructured substrates were prepared as described in^33^ with modifications. Shortly, AgNSSs were obtained by ultrasonic deposition of aqueous diaminsilver (I) hydroxide solution on glass plates under 270–300 °C for 60 min. To obtain diaminsilver hydroxide 0.1 M aqueous sodium hydroxide (Aldrich NaOH, high purity water, Milli-Q, Millipore) was added drop-wise to freshly prepared 0.01 M aqueous silver nitrate solution until complete precipitation of a black-brown silver oxide (I). The prepared silver oxide (I) was thoroughly washed with deionized water and dissolved in a two-fold molar excess of a 10% aqueous ammonia solution resulting in the formation of 0.01 M diaminsilver (I) hydroxide. The obtained transparent complex solution was filtered through Millex-LCR syringe driven filter units (Millipore, 0.45 μm pores). Next, the diaminsilver hydroxide solution was nebulized into mist and 1–5 micrometer droplets were streamed on cover glass under 270 °C. The obtained nanostructured substrates demonstrate plasmonic behavior without applying any additional treatment.

### Mitochondria preparation

Mitochondria were isolated from hearts of male Sprague-Dawley rats, body weight 300–350 g (M&M Taconic, Denmark). The animal studies conformed with the Guide for Care and Use of Laboratory Animals (National Institutes of Health Publication No. 85–23, revised 1996) and Danish legislation governing animal experimentation, 1987, and were carried out after permission had been granted by the Animal Experiments Inspectorate, Ministry of Justice, Denmark. Briefly, after the animal sacrifice heart was isolated and placed immidiately into the ice-cold 1.5% KCl solution (50 ml), washed out from blood and fat and connective tissue were removed with scissors. After that the heart was sliced into 1 mm-sized pieces and then was homogenized on ice in 6 ml of a buffer consisting of (in mM) 10 Tris-HCl, 2 EGTA, 300 sucrose, 2 phenyl-methyl-sulphonylfluoride, aprotinin 10 μg/ml (pH 7.1), using a sequence of two rotating blade homogenizers (10 strokes with Ultra Turrax, Bie&Berntsen, DK; followed by 10 seconds with Polytron PT1200, Holm&Halby, DK) or with glass/teflon potter homogenizer during 3 min. The homogenate was diluted with equal volume of the same buffer and centrifuged at 700 g for 10 min, followed by a centrifugation of the supernatant at 7000 g for 10 min. The pellets representing the mitochondria-rich fraction were washed at 7000 g for 10 min in a combined volume of 8 ml, after that the sedimentation was resuspended in 2 ml of MSTP-buffer (225 mM mannitol, 75 mM sucrose, 20 mM Tris base, 10 KH_2_PO_4_, and 0.5 mM EDTA) resulting in sample 1 that was stored at 4 °C. Concentration of total protein in the obtained mitochondrial sample 1 was 3.5–4.4 mg/ml. SERS experiments were done during first 2–3 h after mitochondria isolation.

### SERS and RS spectra recording

Raman microspectrometer InVia (Renishaw, UK) with 532 nm laser was used for measurements of SERS and RS spectra. Directly before each SERS experiment 30 μl of the mitochondrial sample 1 were diluted 20 times with the physiological phosphate-based solution (composition, mM: 150 KCl, 5 Tris-HCl, 1 EGTA, 5 Na_2_HPO_4_, 1.5 CaCl_2_, pH 7.1, 4 °С) resulting in experimental mitochondrial sample 2. After that in order to supply mitochondrial ETC with electrons and to stimulate ATP synthesis by complex V we added sodium pyruvate (2 mM), sodium succinate (5 mM), ADP (2 mM) and MgCl_2_ (3 mM) into the mitochondrial sample 2. Then 300 μl of the mitochondrial sample 2 were dropped on the AgNSS preliminary placed into a Petri dish with the glass bottom. SERS spectra of mitochondria were recorded by focusing laser light slightly above AgNSS surface using objective x5, NA 0.15 with laser power 1.5 mW per excitation spot with 2–3 μm diameter, accumulation time for each SERS spectrum was 20 s. Control SERS spectra of mitochondria were registered in 30–60 s after the sample placement on AgNSS and after that FCCP (carbonyl cyanide 4-(trifluoromethoxy)phenylhydrazone) or oligomycin (Sigma) in final concentrations 0, 5 and 10 μM, respectively, were added. SERS spectra were recorded within 2 min after FCCP or oligomycin application. The whole SERS experiment with each mitochondrial sample was done within 5–6 min after mitochondria dilution with potassium-phosphate-based buffer and mitochondria placement on AgNSS. Within these time limits we were sure that mitochondria retained their integrity with the functional ETC and the coupling between the electron transport and ATP synthesis. To record SERS spectra of mitochondria with reduced ETC, we added sodium dithionite (SDT) to mitochondrial sample 10–15 min before mitochondria were placed on AgNSS.

To record ordinary Raman spectra the mitochondria suspension was placed directly on the glass bottom of Petri dish. Raman spectra were recorded by focusing laser light inside thick mitochondrial aggregates using objective x63, NA 0.9 with laser power of 1.5 mW per excitation spot with 400–500 nm diameter.

### SERS spectra analysis

SERS and Raman spectra were processed using open source software Pyraman available at http://bitbucket.org/alexeybrazhe/pyraman. Baseline was subtracted in each spectrum. Baseline was defined as a cubic spline interpolation of a set of knots, number and *x*-coordinates of which were selected manually and fixed for all spectra in the study on intervals outside any informative peaks in the spectra, whereas *y*-coordinates of the knots were defined separately for each spectrum as 5-point neighborhood averages of spectrum intensities around the user-specified *x-*position of the knot. Same number and x-positions of knots were used for all the spectra in the study. The parameters for baseline subtraction were chosen after preliminary processing of a randomly chosen set of approximately 40 spectra from different preparations to ensure that all typical baseline variations were taken into account. To compare changes in SERS spectra after FCCP and oligomycin treatment we calculated ratios of maximal intensities of peaks with maxima positions at 748, 1170, 1371 and 1638 cm^−1^. For this purpose maximal intensities of peaks at 748, 1170 and 1371 cm^−1^ were normalized to the maximal intensity of peak at 1638 cm^−1^ giving ratios I_748_/I_1638_, I_1170_/I_1638_ and I_1371_/I_1638_. Calculation of intensity ratios was done after the baseline subtraction.

### Reflectivity measurements

Scattering properties of the fabricated nanostructures were studied using spatially resolved linear reflection spectroscopy. The spectroscopic reflection analysis was performed on a BX51 microscope (Olympus) equipped with a halogen light source, polarizers and a fiber-coupled grating spectrometer QE65000 (Ocean Optics) with a wavelength resolution of 1.6 nm. The reflected light was collected in backscattering configuration using MPlanFL (Olympus) objectives with magnifications × 50 (NA = 0.75). The microscope images (1600 × 1200 pixels) were captured with a LC20 digital color camera (Olympus). The experimental data in Fig. 3D represent the reflectivity determined as the ratio *R*_str_*/R*_ref_, where *R*_str_ is the reflection measured from the structure and *R*_ref_ is the reference spectrum recorded from the reference broadband laser mirror (Edmund Optics, 11 NT64-114) that exhibits an average reflection of 99% between 350 and 1100 nm of light wavelengths.

### SEM and EDX measurements

To perform visualization of AgNSSs Carl Zeiss NVision 40 electron microscope with 7 kV accelerating voltage for SEM was used. 20 kV EDX Oxford Instruments X-Max detector collecting data over 10 000 μm^2^ area was used to provide results of local chemical analysis.

### Numerical model

Our approach is based on the coupled-dipole method^53^ where the dipole moments of nanoparticles are found by solving the following equations


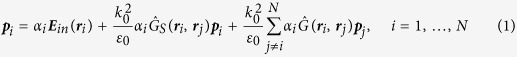


where ***p***_*i*_ and *α*_*i*_ are the dipole moment and the electric dipole polarizability of the *i*-th nanoparticle. ***r***_*i*_ defines its position, and *N* is the total number of particles. ***E***_*in*_ is the external electric field given by the superposition of the incident wave and reflected wave by the silver substrate (without particles), *k*_0_ and *ε*_0_ are the vacuum wave-number and dielectric constant, respectively. The Green tensor 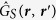 of the system without nanoparticles describes the electric field at the point ***r*** created by the electric dipole at the point ***r***′. 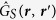 takes into account only fields reflected from the substrate surface, representing a part of the total 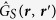. For a single interface system, this tensor can be found in ref. 54. In our model the electric dipole polarizabilities *a*_*i*_ of spherical nanoparticles are taken from Mie theory^55^. Using dipole moments of nanoparticles determined by Eqs. (1), the total electric field outside nanoparticles is calculated by


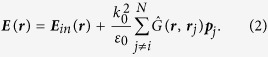


Dielectric permittivity of silver is taken from ref. 56.

## Additional Information

**How to cite this article**: Brazhe, N. A. *et al.* Probing cytochrome *c* in living mitochondria with surface-enhanced Raman spectroscopy. *Sci. Rep.* **5**, 13793; doi: 10.1038/srep13793 (2015).

## Supplementary Material

Supplementary Information

## Figures and Tables

**Table 1:** Assignment of the main peaks in SERS spectra of mitochondria and purified cytochrome *c* (based on refs 13,14,16,18). ΔΦ—potential of the internal mitochondrial membrane.

Mitochondria		Isolated cytochrome *c*		Vibration symmetry	Bond vibration	Sensitivity
SDT-untreated	SDT-treated	Oxidized	Reduced			
1638	1605	1638	1608	B1g, ν10	C_a_C_m_, C_a_C_m_H, C_a_C_b_	Spin state of heme Fe, diameter of porphyrin ring
1585	1545	Presumably 1585, hiding by the peak at 1569	1549	A2g	C_a_C_m_, C_a_C_m_H, C_a_C_b_	Spin state of heme Fe, diameter of porphyrin ring
1565		1569		B1g	C_a_C_m_, C_a_C_b_, C_a_N	Spin and Redox states of heme Fe
1403	1396	1403	1400	B2g	C_a_C_b_, C_b_C_1_	Redox state of heme Fe
1371	1356	1373	1363	A1g, ν4	Symmetric pyrrol half-ring	Redox state of Fe
1313	1311	1317	1316		All bonds of heme *c*	Redox state of heme Fe, ΔΦ
1170	1170	1172	1172	B2g, ν30	Asymmetric pyrrol half-ring	Redox state of heme Fe, ΔΦ
1127	1127	1130	1130	B1g, ν5	C_b_-CH_3_	ΔΦ
748	748	758	758	B1g, ν15	Heme breathing	Redox state of heme Fe, ΔΦ
	690		690			Redox state of heme Fe

Numbering of heme carbon atoms is shown in Supplementary Figure 1.
