# Supplementary material for: Probing cytochrome c in living mitochondria with surface-enhanced Raman spectroscopy
Source: Sci Rep. 2015 Sep 8;5:13793. doi: 10.1038/srep13793 (PMC4561893; doi:10.1038/srep13793)
Supplement: Supplementary Information [file srep13793-s1.doc]

**Supplementary Information**

**Probing cytochrome *c* in living mitochondria with surface-enhanced Raman spectroscopy**

Nadezda A. Brazhe^*^, Andrey B. Evlyukhin, Eugene A. Goodilin, Anna A. Semenova, Sergey M. Novikov, Sergey I. Bozhevolnyi, Boris N. Chichkov, Asya S. Sarycheva, Adil A. Baizhumanov, Evelina I. Nikelshparg, Leonid I. Deev, Eugene G. Maksimov, Georgy V. Maksimov, Olga Sosnovtseva^*^

*^*^corresponding authors* [*nadezda.brazhe@*](mailto:nadiya.brazhe@gmail.com)*biophys.msu.ru and* [*olga@sund.ku.dk*](mailto:olga@sund.ku.dk)


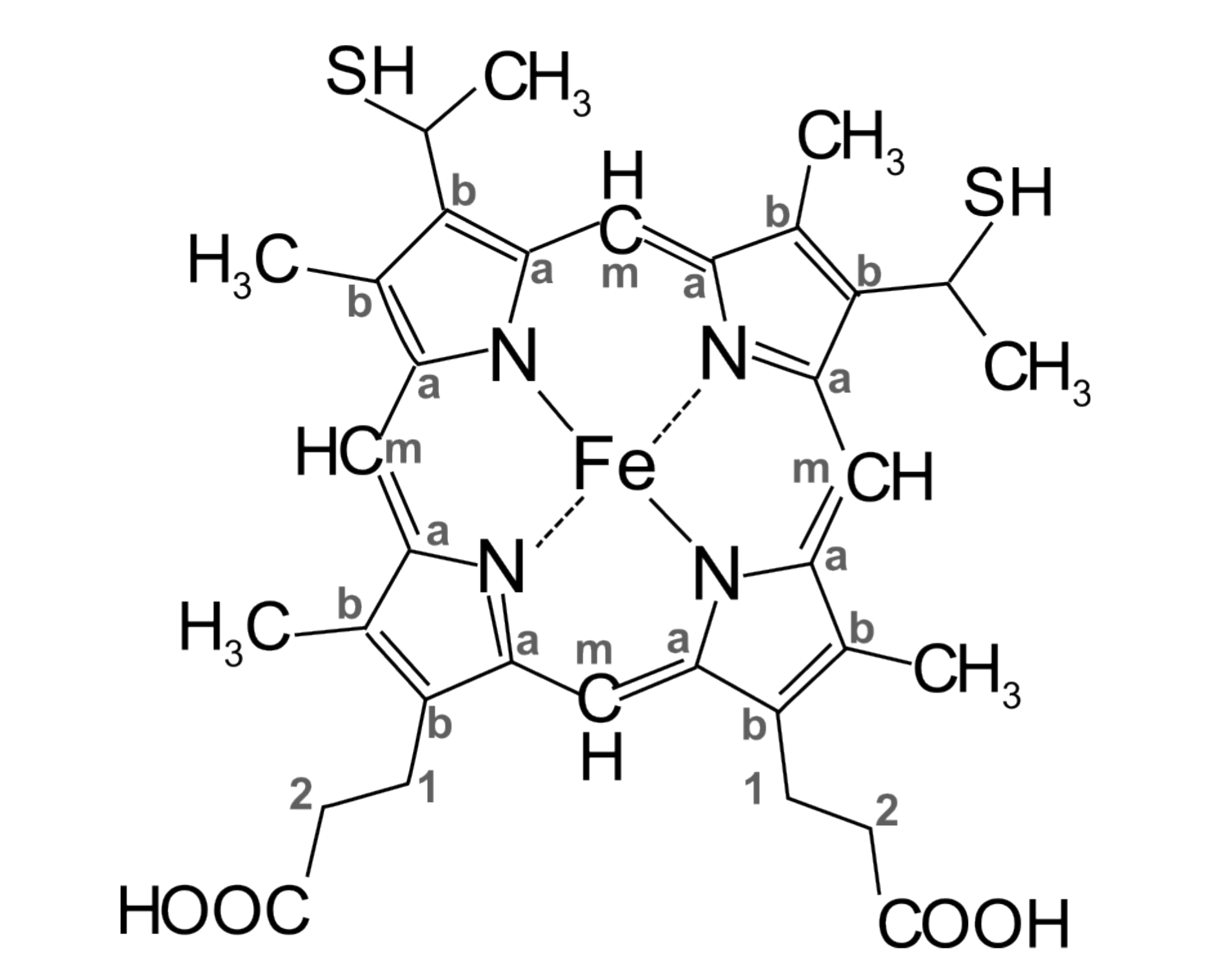


**Supplementary Figure 1.** Structural formula of heme *c*. Numeration of carbon atoms of the porphyrin is shown by small gray letters, the assignment of carbon atoms of side radicals is shown by numbers.


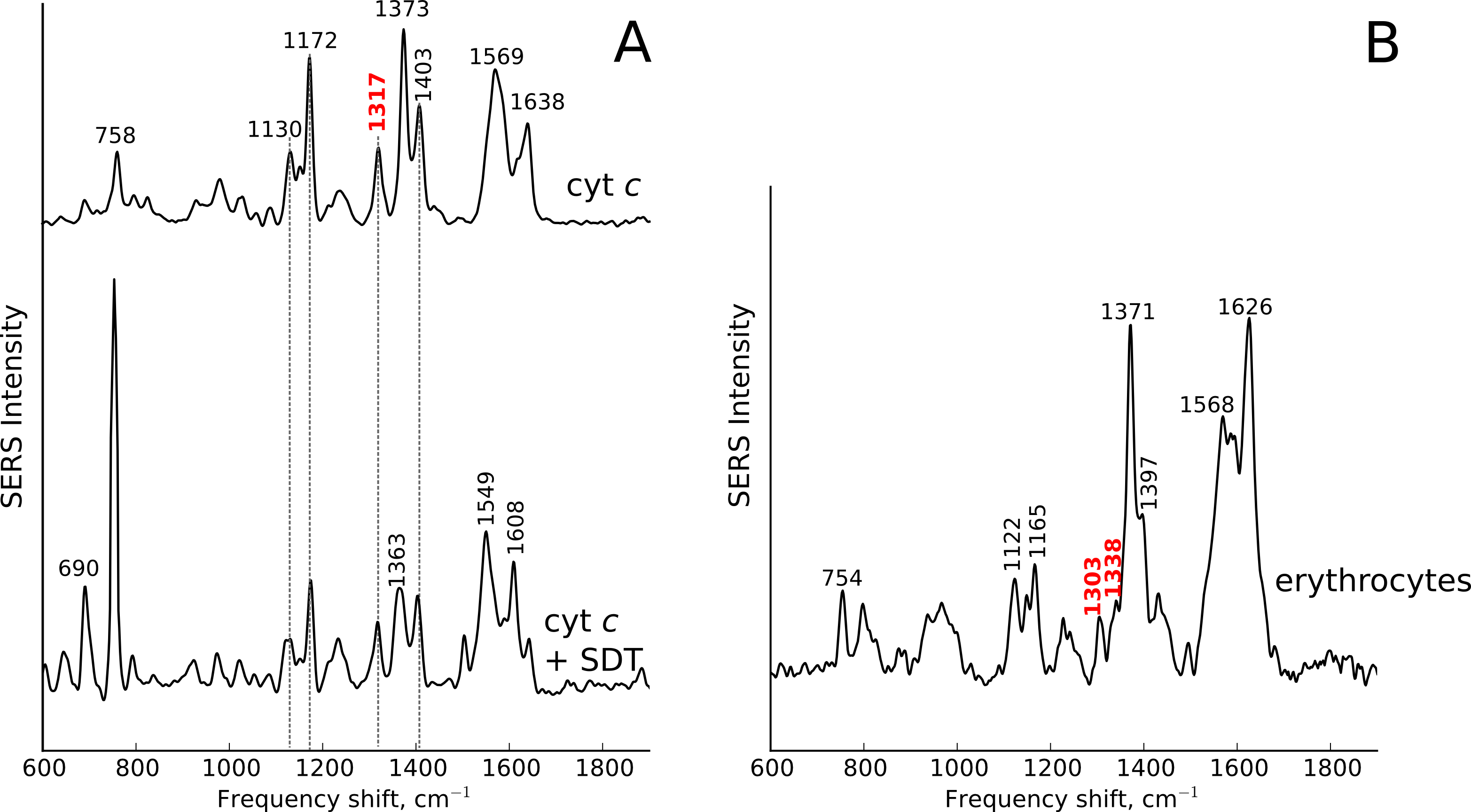


**Supplementary Figure 2.** (A) SERS spectra of purified reduced and oxidized purified cytochrome *c* (1 μM, Sigma) (bottom and top spectra, respectively) mixed with silver colloid in volumetric ratio 1:1. (B) SERS spectrum of oxygenated erythrocyte suspension obtained by 10^4^ times blood dilution placed on AgNSS. SERS spectrum of erythrocytes correspond to the SERS spectrum of *b*-type hemoporphyrin (in this case, oxyhemoglobin). Laser wavelength is 532 nm. The numbers above SERS peaks indicate positions of their maxima. Red colored numbers correspond to the positions of the characteristic peaks of *c*-type heme (1317 cm^-1^, Fig. A) and *b*-type heme (1303 and 1338 cm^-1^, fig. B), respectively.


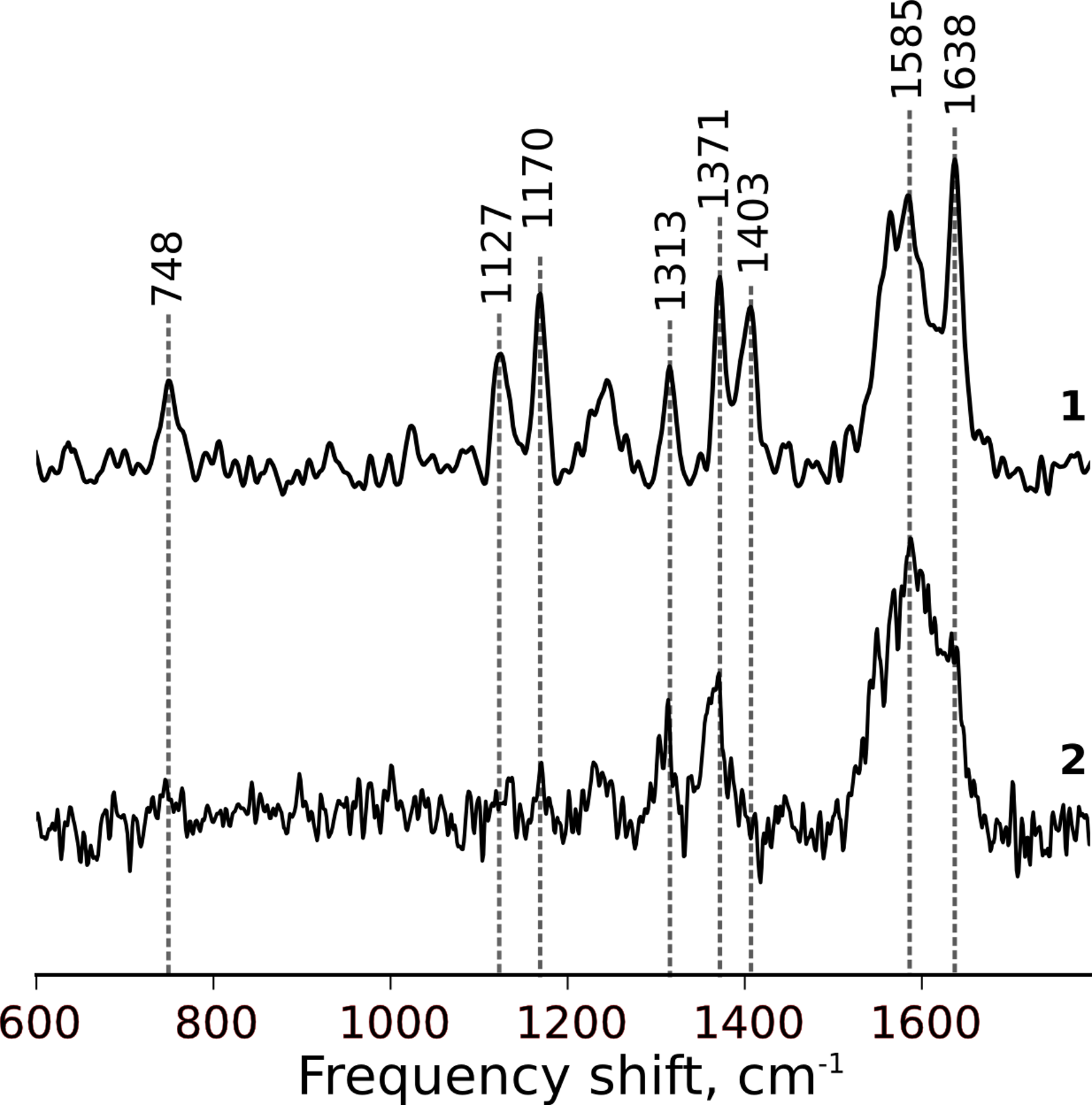


**Supplementary Figure 3.** (1) SERS spectrum of mitochondria (placed on AgNSS with pyruvate, succinate and ADP in physiological buffer) recorded under 1.5 mW excitation power per registration spot of 2 μm diameter, accumulation time 20 s; (2) SERS spectrum of mitochondria recorded under intentionally increased laser power (15mW excitation power per registration spot of 2 μm diameter, accumulation time 40 s, before the spectrum recording mitochondria were illuminated during 3 min). Numbers above vertical gray lines indicate maximum positions of main SERS peaks. Sample excitation with the increased laser power causes peaks' broadening and disappearance of the fine spectrum structure (spectrum 2).

**Registration of excitation spectra of mitochondria fluorescence**

Fluorescence excitation spectra of mitochondria were measured using Fluoromax-4 fluorimeter (Horiba Jobin Yvon, Japan). Fluorescence emission was recordered at 490 nm (the bandwidth of the slit was set to 1 nm), fluorescence excitation was performed in 280-450 nm region (5 nm slit). As in experiments on O_2_ consumption we used two types of mitochondria samples: (1) control mitochondria and (2) mitochondria diluted by potassium-phosphate-based buffer and incubated for 6 minutes on silver nanostructured surfaces and illuminated by 532 nm laser light. For measurements of fluorescence excitation mitochondria suspension with added succinate (5 mM), pyruvate (2 mM), ADP (2 mM) and MgCl_2_ (3 mM) was placed in the quartz cuvette. Excitation spectra of mitochondria autofluorescence were recorded immediately after mitochondria placement into the cuvette and then after addition of FCCP in 1 μM and 20 μM concentrations (Supplementary Figure 4). In both control mitochondria and mitochondria incubated on AgNSS FCCP caused the gradual decrease in the intensity of mitochondria autofluorescence with the most significant changes when excited in 340-370 nm region. This region corresponds to the absorption of NADH (reduced form) and therefore the obtained result indicates the decrease in the amount of NADH [Brandes and Bers, 1996; Wang et al., 2009]. FCCP(or CCCP)-induced decrease in the amount of reduced electron donors and reduced carries in respiratory chain of mitochondria is well-known for isolated mitochondria and mitochondria in cells and tissues and is due to the increased rate of the electron transport in the respiration chain in order to recover Dm_H+_.

Brandes, R., and D. M. Bers. Increased work in cardiac trabeculae causes decreased mitochondrial NADH fluorescence followed by slow recovery. *Biophys. J.* 71:1024 –1035 (1996)

Hsing-Wen Wang, Yau-Huei and Han-Wen Guo. Reduced Nicotinamide Adenine Dinucleotide (NADH) Fluorescence for the Detection of Cell Death. *Anti-Cancer Agents in Medicinal Chemistry,* 9, 000-000 (2009)


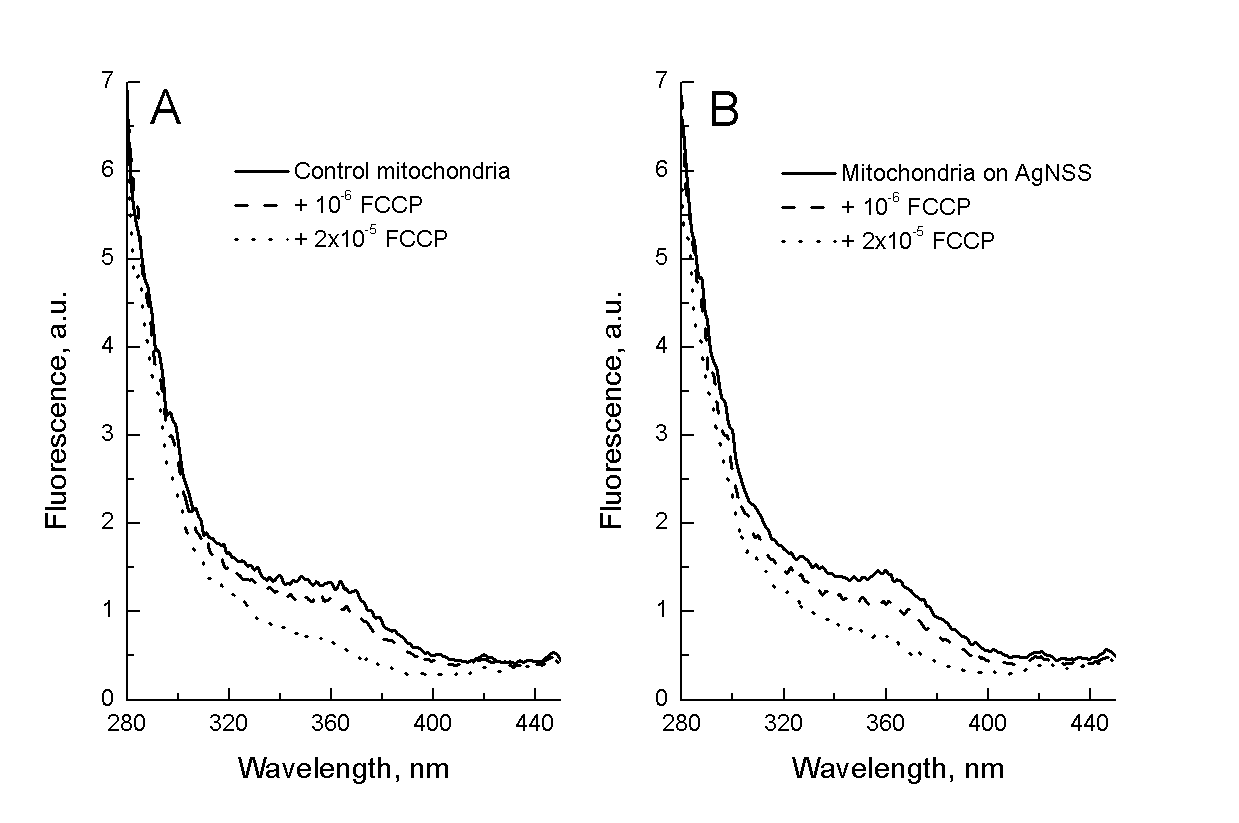
**Supplementary Figure 4.** Excitation spectra of mitochondria autofluorescence measured at 490 nm before FCCP application (solid lines) and after FCCP application in final concentration of 1 and 20 μM (dashed and dotted lines, respectively). Experiments were done on mitochondria kept in MSTP buffer and diluted by potassium-phosphate buffer used in SERS-experiments immediately before registration of the excitation spectra(A) and on mitochondria, diluted by potassium-phosphate buffer and incubated on AgNSS for 6 min under the periodical 532 nm laser illumination (B).

**Measurement of oxygen consumption by mitochondria.**

To study effect of the isolation procedure, potassium-phosphate-based buffer and AgNSS and laser illumination on mitchondria integrity we estimated rate of oxygen consumption by mitochondria after sequential application of ADP and protonophore FCCP. Rate of oxygen consumption by mitochondria was estimated by polarographic analyzer PU-1 (Homel, Belorussia) developed to monitor amount of oxygen in solutions with biological objects. Data transfer to PC was done by means of Metex M-4660 multimeter (South Korea). For this purpose we used two types of mitochondria samples: (1) control mitochondria stored in MSTP-buffer and transferred directly to the polarographic chamber, (2) mitochondria diluted with potassium-phosphate-based buffer used in SERS experiments and incubated for 6 min in chamber with AgNSS bottom (similar to those used in SERS experiments) with the periodic 20 s illumination by 532 nm laser (total illumination time was 60 s, laser power was the same as in SERS experiments). Incubation was done at 25 ^o^C and the chamber was opened to the air. To perform measurement of O_2_ consumption 60 μl of mitochondria were transferred to the 1.5 ml polarographic temperature-controlled (25 ^o^C) sealed chamber with the physiological buffer used in SERS experiments. Buffer was already equilibrated to O_2_. Final amount of mitochondria in polarographic chamber was the same as in SERS experiments. After that we added (1) sodium succinate (5 mM)+ sodium pyruvate (2 mM); (2) ADP (2 mM)+ MgCl_2_ (3 mM); (3) protonophore FCCP (20 μM). All applications were done when the oxygen consumption rate reached the steady state. Number of independent experiments in each mitochondrial group was 2. Supplementary Table 1 shows rate of oxygen consumption (nmol О_2_/mg protein/min) calculated for each group. Data are shown as mean value±SE. It can be seen that ADP and FCCP applications caused the increase in the O_2_ consumption rate that demonstrate existence of the H^+^-gradient and coupling between electron transport and ATP synthesis in mitochondria before FCCP treatment. This is an evidence of the integrity of the mitochondrial membranes in control mitochondria and mitochondria kept under conditions of SERS-experiment. There was no difference between O_2_ consumption by control mitochondria and mitochondria incubated on AgNSS under laser illumination.

**Supplementary Table 1.** Rate of oxygen consumption by control mitochondria and mitochondria, incubated 6 min on the silver nanostructured surface under 1 min of the illumination by 532 nm laser. Rates of O_2_ consumption are shown as nmol О_2_/mg protein/min. Data are shown as mean values±SE, number of independent experiments n=2.

| Application | Mitochondria after 6 min incubation of glass surface | Mitochondria after 6 min incubation in the chamber with AgNSS and 1 min illumination with 532 nm laser |
| --- | --- | --- |
| Mitochondria | 8.5±4.2 | 10.5±5.3 |
| Succinate (5 mM)+pyruvate (2 mM) | 27.8±7.5 | 25.3±8.1 |
| ADP (2 mM) + MgCl_2_ (3 mM) | 95.5±9.5 | 81.1±4.1 |
| FCCP (20 μM) | 178±12.7 | 169±16.7 |


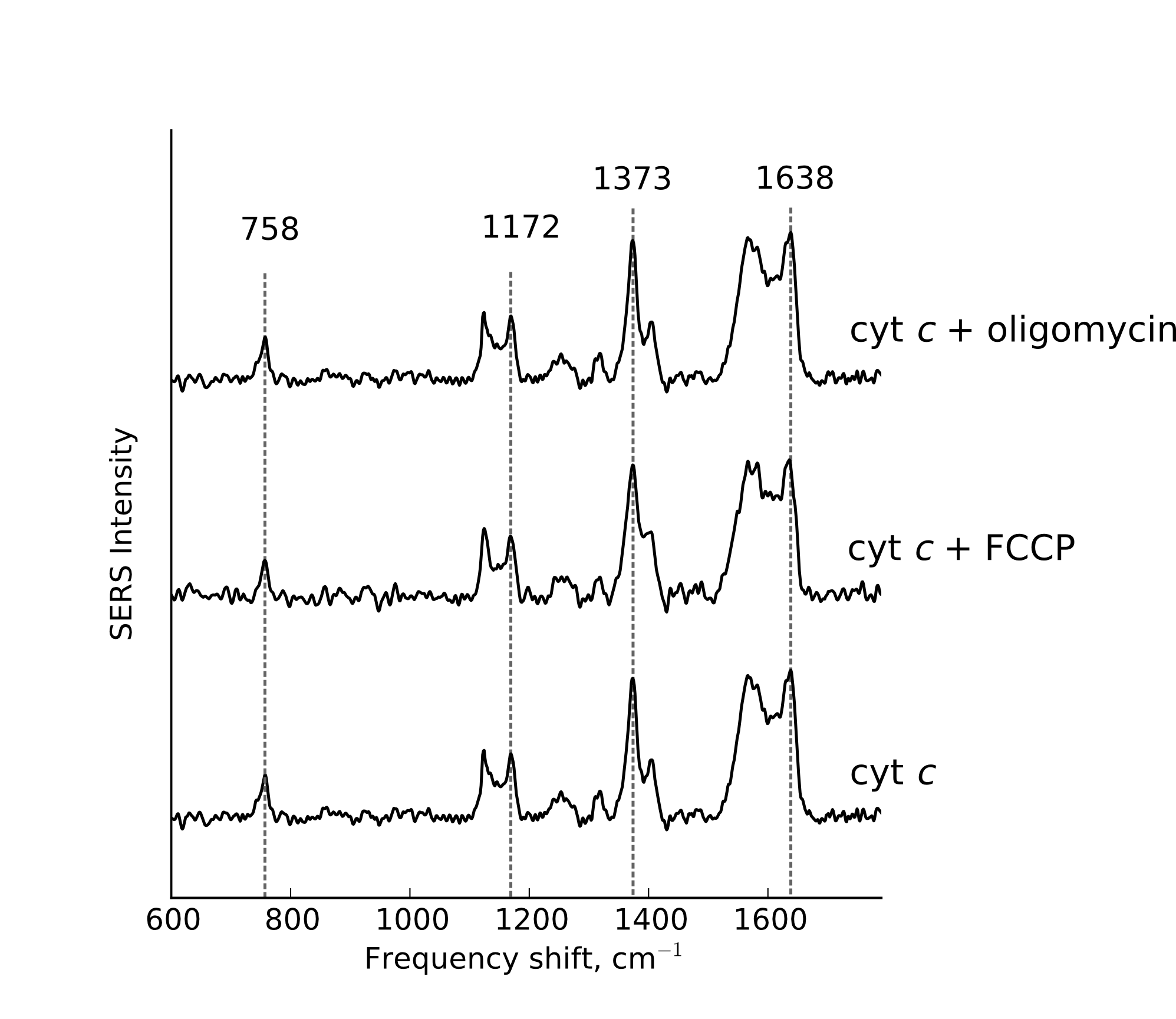


**Supplementary Figure 5.** SERS spectra of purified oxidized cytochrome *c* (1 μM, Sigma) (lower spectrum) and cytochrome *c* after application of FCCP (1 μM) (middle spectrum) and oligomycin (10 μM) (upper spectrum). SERS spectra were recorded after mixing of cytochrome *c* sample with silver colloid in volumetric ratio 1:1. The numbers above SERS peaks indicate positions of their maxima.


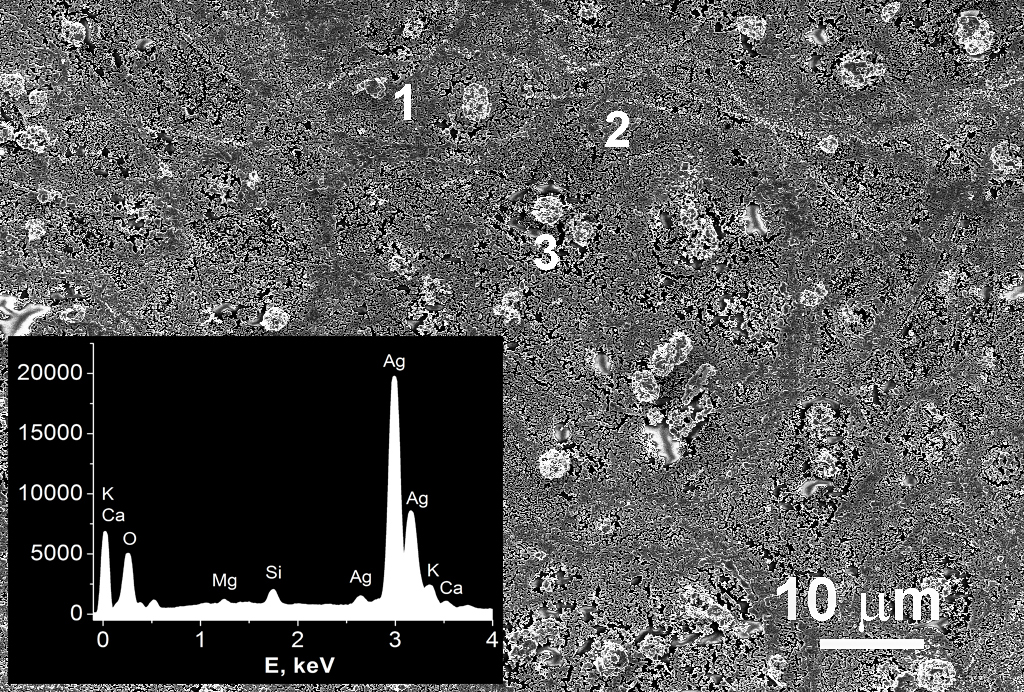


**Supplementary Figure 6.** SEM photograph and local chemical analysis data (black inset) of aerosol deposited silver nanostructured substrates after their successful application for SERS recording of living mitochondria. 1 - silver nanoparticles, 2 – boundaries of silver rings, 3 - residual salts from the mitochondria physiological buffer on the surface of substrate. The inset shows EDX analysis data over square area demonstrating element signals from silica glass (Si, O, K, Ca) and also strong silver peaks (Ag).


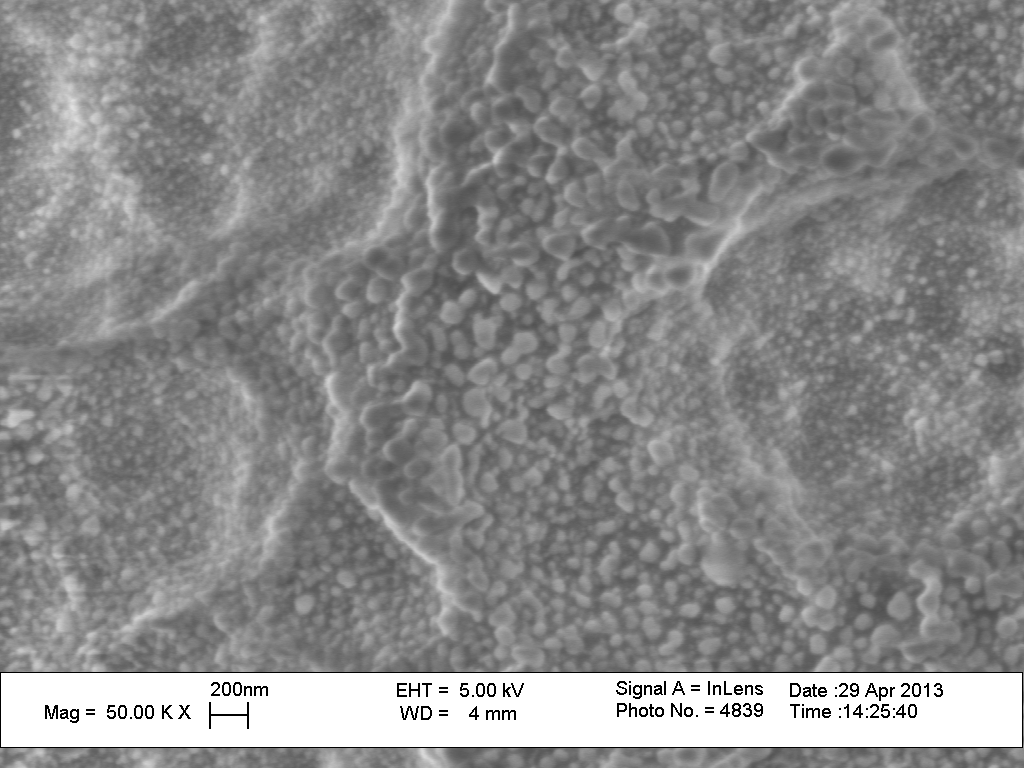


**Supplementary Figure 7.** SEM photograph of magnetron sputtered Ag nanostructured surface consisting of spherical Ag nanoparticles and their clusters without multiscale structure.
